# Knockout of the Carbohydrate Responsive Element Binding Protein Enhances Proliferation and Tumorigenesis in Renal Tubules of Mice

**DOI:** 10.3390/ijms252111438

**Published:** 2024-10-24

**Authors:** Kerrin Hansen, Kristin Peters, Christian K. Burkert, Eric Brose, Diego F. Calvisi, Katrina Ehricke, Maren Engeler, Elisa Knuth, Nils Kröger, Andrea Lohr, Jessica Prey, Jenny Sonke, Padmanabhan Vakeel, Juliane Wladasch, Jenny Zimmer, Frank Dombrowski, Silvia Ribback

**Affiliations:** 1 Institut für Pathologie, Universitaetsmedizin Greifswald, DE-17489 Greifswald, Germany; kristin.peters@med.uni-greifswald.de (K.P.); maren.engeler@med.uni-greifswald.de (M.E.); jessica.prey@med.uni-greifswald.de (J.P.); p.vakeel@gmail.com (P.V.); juliane.wladasch@stud.uni-greifswald.de (J.W.); frank.dombrowski@uni-greifswald.de (F.D.); silvia.ribback@uni-greifswald.de (S.R.); 2 Institut für Pathologie, Universität Regensburg, DE-93053 Regensburg, Germany; diego.calvisi@klinik.uni-regensburg.de; 3 Klinik und Poliklinik für Urologie, Universitaetsmedizin Greifswald, DE-17489 Greifswald, Germany

**Keywords:** Akt/mTOR, clear cell tubules, glycogen, MLXIPL, nephrocarcinogenesis, proliferation, renal cell carcinoma, streptozotocin, tumor development

## Abstract

Glycogen-storing so-called clear cell kidney tubules (CCTs), precursor lesions of renal cell carcinoma, have been described in diabetic rats and in humans. The lesions show upregulation of the Akt/mTOR-pathway and the related transcription factor carbohydrate responsive element binding protein (ChREBP), which is supposedly pro-oncogenic. We investigated the effect of ChREBP-knockout on nephrocarcinogenesis in streptozotocin-induced diabetic and normoglycemic mice. Diabetic, but not non-diabetic mice, showed CCTs at 3, 6 and 12 months of age. Glycogenosis was confirmed by periodic acid schiff reaction and transmission electron microscopy. CCTs in ChREBP-knockout mice consisted of larger cells and occurred more frequently compared to wildtype mice. Progression towards kidney tumors was observed in both diabetic groups but occurred earlier in ChREBP-knockout mice. Proliferative activity assessed by BrdU-labeling was lower in 1-week-old but higher in 12-month-old diabetic ChREBP-knockout mice. Surprisingly, renal neoplasms occurred spontaneously in non-diabetic ChREBP-knockout, but not non-diabetic wildtype mice, indicating an unexpected tumor-suppressive function of ChREBP. Immunohistochemistry showed upregulated glycolysis and lipogenesis, along with activated Akt/mTOR-signaling in tumors of ChREBP-knockout groups. Immunohistochemistry of human clear cell renal cell carcinomas revealed reduced ChREBP expression compared to normal kidney tissue. However, the molecular mechanisms by which loss of ChREBP might facilitate tumorigenesis require further investigation.

## 1. Introduction

With an incidence of about 19/100.000, renal cell carcinoma (RCC) is one of the most common malignancies in the Western world. At localized stages, clear cell renal cell carcinoma (ccRCC), the most common type, is usually amenable to surgical resection and associated with a good prognosis. However, in advanced stages, the prognosis is dismal. Due to demographic change, the number of RCC cases is expected to increase [1]. Therefore, a better understanding of its pathogenesis is essential to develop novel therapies.

An upregulation of the protein kinase B (Akt)/mammalian target of rapamycin (mTOR) pathway is frequently detected in ccRCC in humans, and is associated with related metabolic alterations like increased glycolysis and lipogenesis [2,3]. Upregulation of the Akt/mTOR pathway is also involved in diabetic nephropathy. Patients with long-standing diabetes mellitus have an increased risk of developing different types of tumors, including RCC, and RCCs in patients with diabetes are more often high-grade [4].

Diabetic rats develop circumscribed areas of glycogenosis in the distal kidney tubules, which present morphologically as groups of clear cells (clear cell tubules, CCTs). The CCTs resemble preneoplastic lesions described in other rat models of nephrocarcinogenesis, e.g., after N-Nitrosomorpholine administration [5,6]. The preneoplastic character of rat diabetes-related CCTs has been shown in detail in diabetic rats, induced by intraperitoneal injection of the pancreatic islet toxin streptozotocin, and in autoimmune-diabetic BB/Pfd rats. In long-term experiments, rat CCTs tended to progress to basophilic or cystic lesions and nodules with enlarged cells and increasing nuclear atypia, and finally evolved into adenomas and carcinomas [7]. At the molecular level, preneoplastic lesions and tumors showed an upregulation of the Akt/mTOR pathway and the related transcription factor carbohydrate responsive element binding protein (ChREBP). In a previous work from our group, similar morphologic, metabolic, and molecular alterations were shown in human sporadic CCTs, indicating that they also have a preneoplastic character [8].

ChREBP regulates glycolysis and lipogenesis in response to high glucose in various tissues, including the liver, pancreas, adipose tissue, and kidney, by binding the carbohydrate response element (ChoRE) in the promotor regions of its target genes. ChREBP belongs to the Mondo family of transcription factors, which share an intramolecular glucose-sensing module, the Mondo conserved region, and serve as glucose sensors in various species [9,10]. It has an overlapping role with sterol regulatory element binding protein 1c (SREBP-1c), which upregulates lipogenesis in response to insulin, whereas ChREBP upregulates glycolysis and lipogenesis in response to glucose [11]. ChREBP also seems involved in upregulation of glycolysis in tumor cells (Warburg effect) and acts as a proto-oncogenic mediator via Akt/mTOR, cMyc signaling, and other pathways. Indeed, upregulation of ChREBP has been shown in various tumors and has been associated with malignancy [12,13,14,15]. In a previous project from our group, in the pancreatic islet transplantation mouse model of insulin-induced hepatocarcinogenesis, knockout of ChREBP led to a delayed development of hepatocellular carcinomas. These findings led us to hypothesize that ChREBP might also be pro-oncogenic in the kidney. The present study aimed to investigate the effect of a systemic ChREBP-knockout (ChREBP-KO) on nephrocarcinogenesis in the contexts of normoglycemia and diabetes. CCT, other preneoplastic lesions, and tumors were quantified in kidney tissue.

## 2. Results

Kidney tissue from streptozotocin-induced diabetic and non-diabetic C57Bl/6J wildtype (WT) mice and ChREBP-KO mice used in previous works from our group [16,17] was analyzed.

### 2.1. Body Weight and Blood Glucose Levels

The expected weight loss following diabetes induction was more pronounced in ChREBP-KO mice; mean body weight in diabetic ChREBP-KO mice vs. diabetic WT mice was 18.9 vs. 22.2 g at 3 months (*p* < 0.001, *n* = 65). Body weight of non-diabetic ChREBP-KO mice was slightly lower compared to non-diabetic WT mice at 3 months (30.7 vs. 32.1 g, *p* = 0.0226, *n* = 39), but did not differ between ChREBP-KO and WT in older animals. Non-diabetic ChREBP-KO mice showed lower blood glucose levels of 9.0 and 8.4 mmol/L at 6 and 12 months, compared to 9.7 and 11.8 mmol/L in non-diabetic WT mice (*p* = 0.0362, *n* = 39; *p* < 0.001, *n* = 40). Following diabetes induction, blood glucose levels remained at the intended high level until the end of the observation period. Diabetic ChREBP-KO mice showed slightly lower blood glucose levels than diabetic WT mice at 6 months of age (22.8 mmol/L in diabetic ChREBP-KO vs. 28.4 mmol/L in diabetic WT mice, *p* = 0.0465, *n* = 46). At 12 months, an opposite trend was observed (33.7 mmol/L in diabetic ChREBP-KO vs. 25.3 mmol/L in diabetic WT mice, *p* = 0.0075, *n* = 24).

### 2.2. Clear Cell Tubules in Diabetic Mice

**Figure 1:**
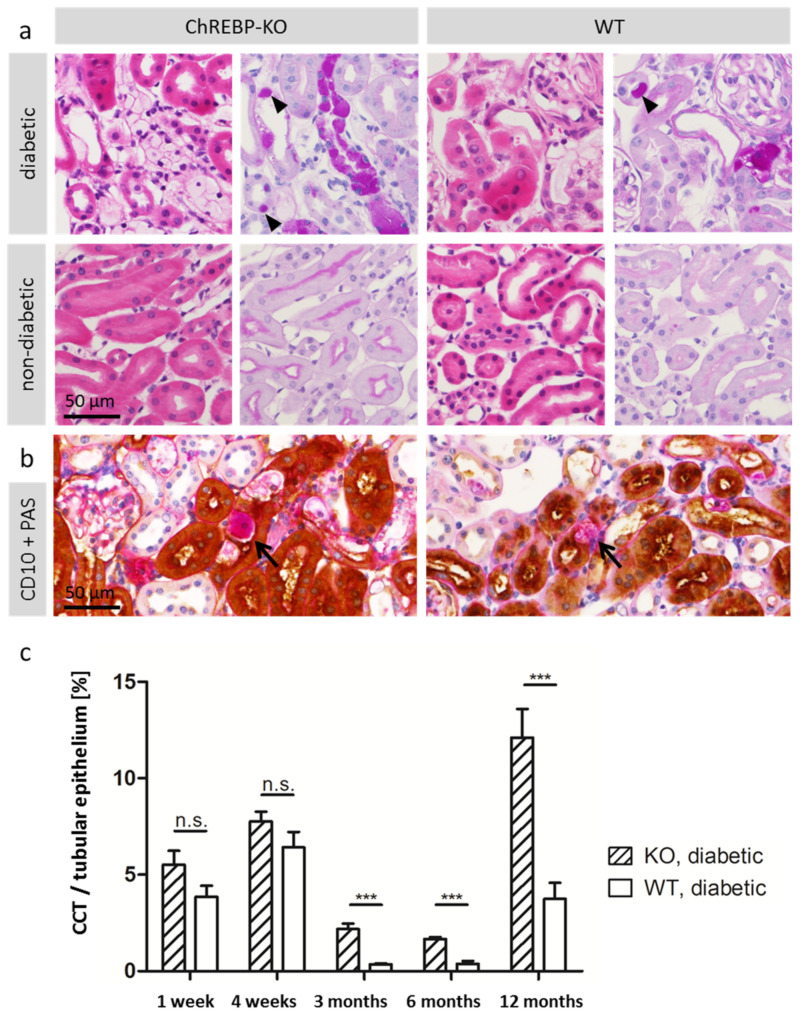
Clear cell tubules (CCTs) in diabetic mice. (**a**) Representative micrographs of H&E-stained kidney sections (left side of each column) show groups of cells with clear cytoplasm in the kidney tubules of diabetic ChREBP-KO and WT mice. The corresponding PAS reaction (right side of each column) is strongly positive in CCTs, indicating glycogenosis. Note the larger clear cells in ChREBP-KO, contrasting small CCTs in WT. Arrowheads highlight glycogen-storing nuclei. (**b**) Co-staining of CD10 and PAS shows glycogen accumulation in proximal tubule cells, indicated by PAS positivity (arrows). (**c**). The graph quantifies PAS-positively stained areas in randomly selected kidney sections of *n* = 233 mice. Data are represented as mean + SEM. *p*-values < 0.001 are indicated by three black asterisks. *p*-values > 0.05 are indicated as n.s. (not significant).

**Figure 2:**
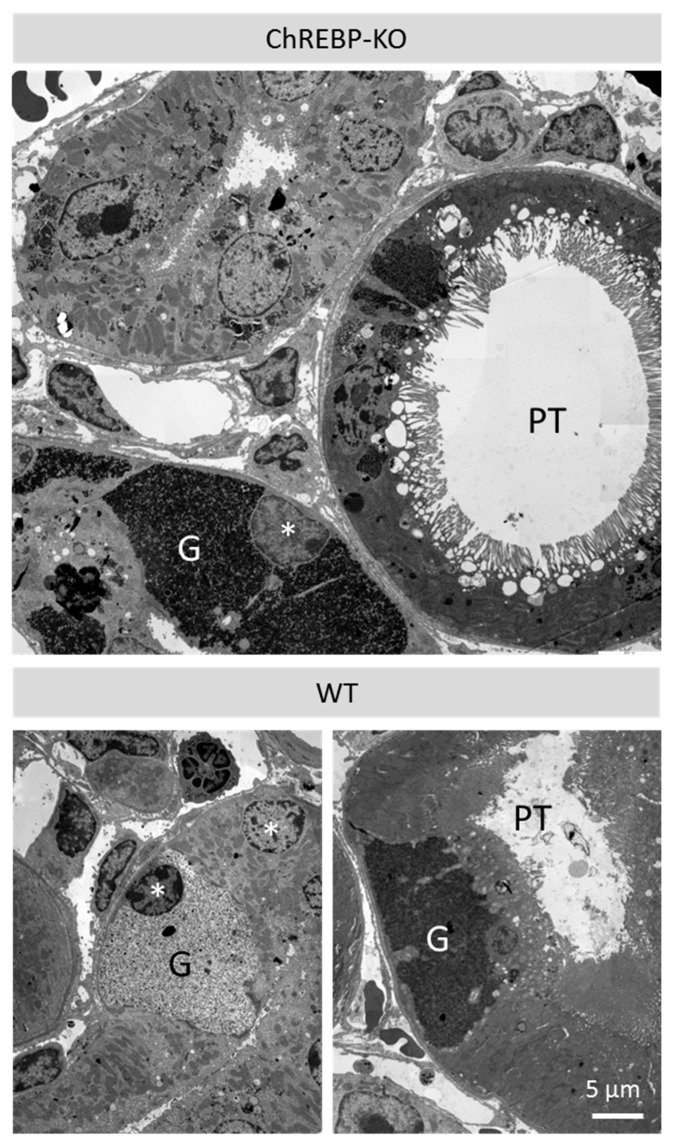
Ultrastructure of CCTs. Transmission electron microscopy (TEM) reveals densely packed α-glycogen (G) particles in the cytoplasm of tubule epithelial cells. Glycogen accumulation was present mainly in distal tubules and also in proximal tubule (PT) cells. White asterisks mark nuclei of distal tubule cells.

Diabetic WT and ChREBP-KO mice developed so-called clear cell tubules (CCTs), groups of tubular epithelial cells with clear cytoplasm in H&E staining, which were strongly positive in periodic acid schiff PAS reaction, indicating glycogen accumulation (Figure 1a). In kidney tubules of WT mice, clear cells were not larger than normal tubular epithelial cells, whereas in ChREBP-KO mice, CCTs presented as prominent groups of enlarged cells. Nuclear glycogen accumulation was occasionally observed in kidney tubules of diabetic animals in both groups (arrowheads in Figure 1a). No CCTs or glycogen-storing nuclei were observed in non-diabetic animals of both genotypes. In transmission electron microscopy (TEM), CCTs presented as enlarged cells with a cytoplasm densely packed with α-glycogen particles (Figure 2). Interestingly, glycogen accumulation was not only found in the distal tubules, but also in the proximal tubules. This finding was confirmed by co-staining of PAS and the proximal tubule marker CD10 (Figure 1b). Differences in CCT size and frequency were quantified morphometrically as the proportion of PAS-positive staining to the whole tubular area in randomly selected sections of kidney tissue. At all observed time points, the volume fraction of CCT was higher in the ChREBP-KO group than WT. It was highest in diabetic ChREBP-KO animals at 12 months of age (12.1 Vol% in KO vs. 3.7 Vol% in WT mice, *p* < 0.0001, *n* = 24; Figure 1c).

### 2.3. Proliferative Activity in the Kidney Tubules

**Figure 3:**
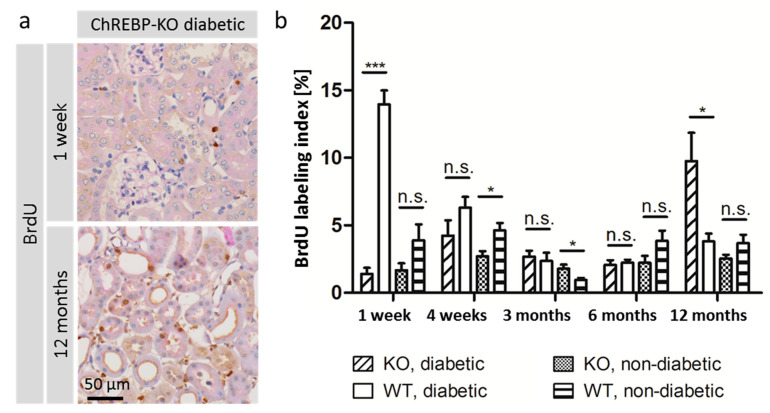
Proliferative activity in the kidney tubules. Proliferative activity was assessed by 24 h BrdU labeling. (**a**) The microscopic images give examples of BrdU staining at 1 week and 12 months after diabetes induction. (**b**) The graph shows the BrdU labeling index (percentage of BrdU-positive tubule epithelial cell nuclei) measured in 15 randomly selected fields of view in 2 kidney sections of *n* = 413 mice. Data are represented as mean + SEM. *p*-values < 0.05 are indicated by one asterisk. *p*-values < 0.001 are indicated by three black asterisks. *p*-values > 0.05 are indicated as n.s. (not significant).

Proliferative activity was assessed using the 24 h BrdU labeling index. The proliferation rate was higher in diabetic animals compared to non-diabetic animals of the same genotype at 4 weeks, 3 months, and 12 months after diabetes induction. Compared to diabetic WT mice, kidney tubules of diabetic ChREBP-KO mice showed a markedly reduced proliferation rate at 1 week (1.4% in KO vs. 14.0% in WT, *p* = 0.0002, *n* = 20), but increased proliferative activity at 12 months (9.8% vs. 3.8%, *p* = 0.0140, *n* = 20). In non-diabetic animals, genotype had no clear effect on proliferative activity in the kidney tubules (Figure 3a,b).

### 2.4. Progression of CCT to Renal Adenomas and Carcinomas

**Figure 4:**
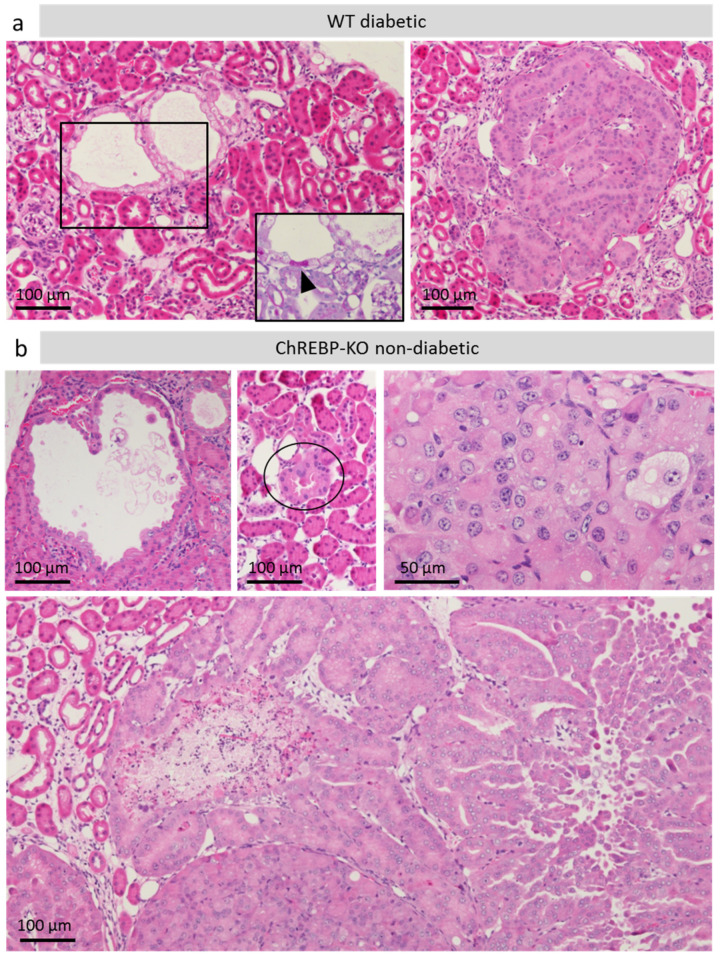
Preneoplastic lesions and kidney tumors. The panel shows micrographs of H&E-stained sections of preneoplastic lesions and tumors observed in diabetic WT and non-diabetic ChREBP-KO mice. (**a**) Renal adenomas present as cystic (**left**) or solid lesions (**right**). Corresponding PAS reaction to the marked section is shown in the box. The arrowhead highlights a small PAS-positive area of glycogenosis. (**b**) Non-diabetic ChREBP-KO mice show cystic and solid preneoplasias and tumors resembling those seen in diabetic animals. The circle marks a preneoplastic lesion with enlarged cells with basophilic cytoplasm. The upper right picture shows nuclear atypia in an advanced lesion. The lower picture shows a renal cell carcinoma.

Beginning at 3 months of age, CCTs in both diabetic groups showed an increase in size associated with loss of PAS-positivity, indicating loss of glycogen. However, in some advanced lesions, a few cells remained PAS-positive. The lesions were either cystic or solid with a basophilic cytoplasm (Figure 4a). Sometimes, transitions between the two growth patterns were seen. The lesions showed nuclear atypia and, in some cases, areas of necrosis, indicating a neoplastic character. Table 1 shows percentages of animals with renal neoplasms, classified by size. A renal cell adenoma was defined as a circumscribed area, measuring ≥ 0.1 mm, in which (1) cells were enlarged compared to surrounding tubular epithelial cells, (2) showed enlarged nuclei and (3) fulfilled at least one of the two morphologic criteria: cystic growth, basophilic cytoplasm. If a lesion fulfilled the criteria for an adenoma and in addition measured ≥ 1 mm in largest diameter and showed areas of necrosis (increased eosinophilia in H&E stain, karyolysis), it was classified as a renal cell carcinoma.

### 2.5. Accelerated Tumor Development in Diabetic ChREBP-KO and Renal Cell Neoplasms in Non-Diabetic ChREBP-KO Mice (Figure 4b, Table 1)

Renal cell neoplasms occurred in both diabetic WT and ChREBP-KO mice, but seemed to develop earlier in ChREBP-KO mice. At 3 months of age, 4% of diabetic WT and 10% of diabetic ChREBP-KO mice presented with one or more renal cell neoplasms of any size. At 6 months, 20% of diabetic WT mice and 25% of ChREBP-KO mice showed one or more renal cell neoplasms. A total of 100% of diabetic WT and 60% of diabetic ChREBP-KO mice showed tumors at 12 months (*p* = 0.0166, *n* = 25). To objectify the suspected trend towards earlier tumor development in diabetic ChREBP-KO mice compared to WT, tumor-free survival was compared using a survival curve. The number of animals without preneoplastic lesions or tumors seemed to decline faster in diabetic ChREBP-KO. However, statistical analysis with the Gehan–Breslow–Wilcoxon test did not show a significantly increased risk of developing tumors for the diabetic ChREBP-KO group compared to WT (Supplementary Figure S1).

Surprisingly, renal cell neoplasms were frequently observed in non-diabetic ChREBP-KO mice, whereas no tumors occurred in non-diabetic WT mice. At 3, 6, and 12 months of age, 26.3% (vs. 0% in WT, *p* = 0.02, *n* = 39), 31.6% (vs. 0% in WT, *p* = 0.02, *n* = 39), and 61.9% (vs. 0% in WT, *p* < 0.0001, *n* = 41) of non-diabetic ChREBP-KO mice showed one or more neoplasms, including RCC. Morphologically, the tumors strongly resembled those seen in diabetic animals. Some showed a basophilic and some a cystic appearance. Cellular atypia was always present, and necrosis was sometimes present. However, no CCTs occurred in non-diabetic ChREBP-KO mice. Therefore, tumors seem to emerge spontaneously. In many cases, multiple tumors occurred.

### 2.6. Immunohistochemical Analysis of Tumors in ChREBP-KO Mice (Figure 5)

In tumors of diabetic and non-diabetic ChREBP-KO mice, the lipogenic enzymes fatty acid synthase (FASN) and acetyl-CoA carboxylase (ACAC) were upregulated compared to adjacent normal kidney tissue. The glycolytic enzyme pyruvate kinase M2 (PKM2) was highly expressed in tumors and morphologically normal distal tubules of diabetic and non-diabetic ChREBP-KO mice. Insulin receptor substrate 1 (IRS1) was highly expressed in tumors and in normal proximal and distal tubules of both groups. The downstream effectors of the Akt/mTOR-signaling pathway 4E-BP1 and RPS6 were upregulated in tumors of diabetic and non-diabetic ChREBP-KO mice. Furthermore, tumors of both groups showed upregulated thioredoxin interacting protein (TXNIP). FASN was higher in normal kidney tissue of ChREBP-KO mice compared to WT mice. There were no differences in the expression of ACAC, PKM2, IRS1, 4E-BP1, pRPS6, or TXNIP in normal kidney tissue between the two genotypes (Supplementary Figure S2). Using immunohistochemistry, we observed strong nuclear positivity for cyclin D1 in renal tumors of non-diabetic ChREBP-KO mice. However, cyclin D1 was also strongly expressed in normal tubular epithelium of diabetic WT mice and less in kidney tubules of non-diabetic ChREBP-KO and WT mice. Tumor cells of non-diabetic ChREBP-KO mice showed strong cytoplasmic positivity for yes-associated protein (YAP). The epithelial–mesenchymal transition (EMT) markers snail, slug, and vimentin were not upregulated in tumors of ChREBP-KO mice, while E-cadherin expression was present (Supplementary Figure S3).

### 2.7. ChREBP Expression in Human RCCs

To investigate whether ChREBP expression is altered in human ccRCCs, we performed immunohistochemistry for ChREBP on FFPE-sections of 31 ccRCCs, 9 of which were graded as G1, 10 as G2, and 12 as G3 according to WHO/ISUP by experienced pathologists (Frank Dombrowski or Silvia Ribback). Tumor tissue was detected by comparison with H&E-stained sections. Nuclear ChREBP expression in tumors was compared to that in adjacent unaltered kidney tissue on the same slide. It was scored according to staining intensity and percentage of positive cells as described in detail in the methods section. ChREBP expression was significantly reduced in tumor vs. normal kidney tissue (score 2 vs. 7.8, *p* < 0.0001, *n* = 31) (Supplementary Figure S4a–c). An upregulation of ChREBP was not observed in any of the 31 ccRCCs. ChREBP expression did not correlate with grading, tumor size, patient age, or body mass index (Supplementary Figure S4d).

## 3. Discussion

In the present work, we investigated the effect of a systemic ChREBP-KO on the development of CCTs and tumorigenesis in streptozotocin-induced diabetic and non-diabetic mice. In humans, the increased risk of developing various types of cancer has been shown predominantly in type II diabetes. However, some studies also show an increased risk in diabetes mellitus type I [18,19,20]. The increased risk of developing tumors in diabetes mellitus might be due to an upregulation of the proliferative pathway of insulin signaling in the setting of hyperglycemia and insulin resistance [21]. However, in rats, the development of CCTs and kidney tumors seems to depend more on the severity of hyperglycemia than the mode of diabetes induction [7]. Therefore, to avoid the unnecessary use of animals, we used kidney tissue of mice injected with streptozotocin in previous experiments [17], resulting in an insulin deficiency, instead of using a mouse model of type II diabetes mellitus like diet-induced obesity (DIO) mice or ob/ob mice [22,23,24,25].

The expected body weight loss following diabetes induction was more pronounced in ChREBP-KO than in WT mice. ChREBP-KO mice seem to not tolerate insulin deficiency like WT mice, possibly because of a mild insulin resistance described before in ChREBP-KO [26]. A reduced body weight might result from a reduced expression of lipogenic enzymes [26]. However, in non-diabetic animals, body weight did not differ significantly between the two genotypes. Non-diabetic ChREBP-KO mice showed slightly lower blood glucose levels than WT controls, whereas Iizuka et al. described higher blood glucose levels in ChREBP-KO mice compared to WT mice at 7 weeks of age [26].

For the first time, we demonstrate that streptozotocin-induced diabetes leads to the formation of glycogen-storing CCTs in the mouse kidney. These CCTs closely resemble those found in diabetic rats and sporadic cases in humans [8]. Proximal tubule injury following streptozotocin administration in mice has been described before [27], but was not associated with CCT formation in the subacute phase. We did not observe histological signs of tubular injury such as significant flattening of tubular epithelial cells, loss of the brush border or interstitial fibrosis.

As described in the rat model, CCTs progressed over several months and evolved into renal adenomas and carcinomas. Therefore, a common mechanism of diabetes-induced nephrocarcinogenesis in mice, rats, and some cases of kidney tumors in humans seems likely. Interestingly, CCTs in mice were not restricted to the distal tubule as in rats and humans, but also occurred in the proximal tubule. This finding might be explained by the expression of Glucose-6-phosphatase in the proximal, but not distal tubules of rats and humans [28]. In mice, morphological differences between the proximal and distal tubules are less marked [29]. However, to our knowledge, there is no evidence for a different distribution of enzymes of glycogen metabolism.

The knockout of ChREBP seems to increase glycogenosis, leading to more and larger CCT formation. Furthermore, diabetic ChREBP-KO mice showed increased proliferative activity in the kidney tubules at 12 months of age. Contrary to our expectations, we observed a trend towards earlier occurrence of pre-neoplastic lesions and tumors in diabetic ChREBP-KO compared to diabetic WT mice, although experiments with larger case numbers would be necessary to verify this effect. The observed transitions from CCTs to advanced lesions and tumors strongly suggest that CCTs are precursor lesions of RCC, as shown in rats [7]. The finding of more and advanced CCTs in ChREBP-KO mice fits an earlier occurrence of RCC, but an explanation for increased glycogenosis in ChREBP-KO mice is missing. More severe hyperglycemia seems very unlikely, because blood glucose levels of diabetic animals did not differ significantly between the two genotypes. Nevertheless, it cannot be ruled out that a reduced glucose tolerance due to reduced expression of glycolytic and lipogenetic enzymes in the liver [26] leads to peaks in blood glucose levels not recorded by the weekly blood glucose measurements performed in our experiment. Another explanation for increased glycogenosis in the kidney tubules of ChREBP-KO mice might be an impaired delivery of glucose from tubule epithelial cells to the blood due to reduced activity of the enzyme glucose-6-phosphatase (G6Pase), which is induced by ChREBP [30]. A reduced G6Pase activity increases glycogenosis in the liver of ChREBP-KO mice [13]. The effect might not be restricted to the liver, leading to increased glycogenosis in the kidney tubules. However, earlier tumor development in diabetic ChREBP-KO mice is not necessarily a consequence of increased glycogenosis due to metabolic alterations. The possibility of a kidney-specific tumor-suppressive function of ChREBP will be discussed below.

Our second major finding is the unexpected and undescribed spontaneous development of pre-neoplastic lesions and tumors in the kidneys of non-diabetic ChREBP-KO mice. Mice often revealed multiple tumors in both kidneys. Tumors of diabetic WT and ChREBP-KO mice and normoglycemic ChREBP-KO mice showed remarkable morphological and molecular similarities. Basophilic or cystic pre-neoplastic lesions were present in all three groups, but in non-diabetic ChREBP-KO mice, glycogenosis was not present. Tumors of diabetic and non-diabetic ChREBP-KO mice showed an upregulation of the lipogenic enzymes FASN, ACAC, and the glycolytic enzyme PKM2 even in the absence of ChREBP. Upregulation of lipogenesis might be mediated via SREBP-1c by upregulation of Akt/mTOR downstream insulin receptor signaling [31]. Indeed, IRS1 was strongly expressed in the tumors and adjacent kidney tissue, while upregulation of 4E-BP1 and RPS6 indicates activation of the Akt/mTOR pathway in tumors [32]. However, if an underlying factor of tumorigenesis was increased activation of proliferative pathways by altered insulin signaling in ChREBP-KO mice [21], one would expect to see differences between tumor development in normoglycemic mice and those with streptozotocin-induced insulin deficiency. Furthermore, proliferative activity in the kidney tubules was not altered in non-diabetic ChREBP-KO mice. In immunohistochemical analysis, we could not detect differences in the expression of lipogenetic and glycolytic enzymes between normal kidney tissue of diabetic and non-diabetic WT and ChREBP-KO mice. Our available data do not provide evidence of a metabolic effect leading to spontaneous kidney tumor development in ChREBP-KO mice. Therefore, the possibility of an unknown kidney-specific tumor-suppressive function of ChREBP must be considered.

Not only the Akt/mTOR pathway but also other pro-oncogenic or tumor-suppressive pathways have been related to ChREBP. In this context, most published works dealing with the role of ChREBP in tumorigenesis indicate that it has a proto-oncogenic function [12,13,14,15], but a few studies also propose a tumor-suppressive role.

In pancreatic β-cells, ChREBP promotes cell cycle progression by upregulating cell cycle regulators in response to glucose, linking metabolic state and proliferation [33]. In contrast to that, a study by Zang et al. provides some evidence that ChREBP acts as a tumor suppressor by binding to cyclin D1 in gastric cancer [34]. In our experiment, upregulation of cyclin D1 was observed not only in renal tumors of ChREBP-KO mice but also in kidney tissue of diabetic WT mice. One might speculate that upregulation of cyclin D1 following loss of ChREBP mimics the proliferation seen in kidney tubules of diabetic mice. However, further research is needed to verify such a mechanism.

In a study by Jiang et al. on non-small cell lung carcinoma cells, suppression of ChREBP and its downstream metabolic targets was sufficient to increase EMT [35]. However, in our study, immunohistochemical analysis did not show an upregulation of the EMT markers used in the work by Jiang et al., and metastases did not occur. Therefore, it seems unlikely that EMT is a contributing factor to increased tumor growth in ChREBP-KO mice.

The tumor-suppressive Hippo pathway, a negative regulator of cell growth and survival, also interacts with ChREBP. When Hippo signaling is active, its terminal effector YAP binds to Taz and is degraded in the cytosol. When the Hippo pathway is inactivated, YAP is translocated to the nucleus to regulate proliferation. A recent study by Shu et al. showed that YAP binds to ChREBP to regulate glucose metabolism in the liver and that Hippo signaling suppressed ChREBP activity [36]. However, to our knowledge, the effect of a ChREBP-knockout on Hippo signaling is still unknown. YAP overexpression has been shown in various malignancies [36]. In our study, enhanced YAP positivity in tumors of ChREBP-KO mice was predominantly cytosolic, which might indicate reduced degradation of YAP.

A study by Zeng et al. suggests a tumor-suppressive function of ChREBP rather than a pro-oncogenic role. The authors showed that ChREBP promotes the differentiation of leukemia-initiating cells (LICs) via the TXNIP/RUNX1 pathways [37]. In contrast to their findings of downregulated TXNIP in ChREBP-null LICs, we observed an upregulation of TXNIP in tumors of ChREBP-KO mice.

In obese patients with intervertebral disc degeneration, ChREBP seems to act pro-apoptotically by activating expression of the pro-apoptotic genes Puma and Bax [38].

The hypoxia-inducible factor 1α (Hif1α) is frequently upregulated in renal cell carcinoma, usually following a loss of function of the Von Hippel–Lindau tumor suppressor (VHL) [3]. Hif1α is also activated in response to high glucose via ChREBP [39], which aligns with an increased risk of developing RCC in diabetes mellitus but makes the finding of increased tumorigenesis in ChREBP-KO mice more surprising.

Taken together, it seems likely that ChREBP fulfills either pro-oncogenic or tumor-suppressive functions depending on the tissue type. A limitation of our study is that the systemic ChREBP-KO model does not allow us to distinguish between a metabolic effect caused by the loss of ChREBP in the liver and a kidney-specific tumor-suppressive function of ChREBP. However, our findings provide the basis for future research in a cell-specific knockout model, which could clarify the molecular mechanisms by which ChREBP-KO leads to ‘spontaneous’ development of kidney tumors.

In 31 cases of ccRCC in humans, ChREBP appears to be downregulated in the tumor compared to unaltered kidney tissue. Based on these data, one might speculate that ChREBP-loss also plays a role in a subset of human RCCs. However, we cannot fully rule out fixation artifacts in the glycogen-rich tumor tissue.

Overall, our findings of increased glycogenosis and accelerated tumorigenesis in diabetic ChREBP-KO compared to WT mice indicate that ChREBP acts as a tumor suppressor rather than a pro-oncogenic mediator in diabetes-induced nephrocarcinogenesis. Spontaneous development of kidney tumors in normoglycemic ChREBP-KO mice strongly suggests a previously unknown tumor-suppressive function of ChREBP in the kidney.

## 4. Methods

### 4.1. Mice Experiments

In this study, all animals received humane care according to the criteria outlined in the “Guide for the Care and Use of Laboratory Animals”, prepared by the National Academy of Sciences and published by the National Institutes of Health (NIH publication 86-23 revised 1985). Animal experiments were approved by the Animal Policy and Welfare Committee of the Universitaetsmedizin Greifswald, Germany (LALLF-MV Rostock, Germany, ref. no. 7221.3-1-012/16). Housing of the animals followed the guidelines of the Society for Laboratory Animal Service and the German Animal Protection Law. The study was carried out in compliance with the relevant guidelines and regulations, including the ARRIVE guidelines.

Kidney tissue was obtained from previous experiments [16,17]. Highly inbred 9-week-old male C57Bl/6J wildtype (WT, ChREBP^+/+^) and ChREBP-knockout (ChREBP-KO, ChREBP^−/−^) mice (*n* = 332, body weight > 20 g; Charles River Laboratories, Sulzfeld, Germany) were matched to 20 groups (listed in Table 2).

### 4.2. Diabetes Induction

The mice received a single intraperitoneal dose of streptozotocin (180 mg/kg body weight; Zanosar^®^, Sigma-Aldrich, Darmstadt, Germany) [16,17]. One week following induction, mice with a blood glucose level > 16.7 mmol/L were defined as diabetic. Animals with blood glucose levels > 30 mmol/L that showed signs of deterioration of general conditions, such as severe weight loss, rough fur, apathetic behavior, or hunchbacked posture, received subcutaneous implantation of an insulin stick (“LinBit”, ~0.1 U/24 h; Linshin Canada Inc., Toronto, ON, Canada).

WT and ChREBP-KO control groups did not receive intraperitoneal injection of streptozotocin and remained normoglycemic.

### 4.3. Application of the Nucleoside Analogue 5-Bromo-2′-Deoxyuridine (BrdU)

One week before sacrifice, half of the animals were anaesthetized, and osmotic mini pumps (Osmotic pump model 2001, Charles River Laboratories, Sulzfeld, Germany) filled with BrdU in sodium chloride solution (0.6 mg/d; Sigma-Aldrich, Darmstadt, Germany) were subcutaneously implanted between the scapulae.

### 4.4. Tissue Processing

Animals were sacrificed under anesthesia (ketamine/xylazine 400/40 mg/kg body weight) after 1 week, 4 weeks, 3, 6 and 12 months. Tissue was perfused with 4% dextran and 0.5% procaine hydrochloride in Ringer solution (pH 7.4) via cannulization of the aorta with a 23-gauge needle. The left kidney was clamped at the artery, removed, and frozen in liquid nitrogen-cooled isopentane (2-methyl-butane). The right kidney and remaining tissue were perfused with a fixation mixture of 0.5% glutaraldehyde, 3% paraformaldehyde and 4% dextran in Ringer solution (pH 7.4).

The right kidney was excised and cut into slices, some embedded in paraffin and some pieces fixed in glutaraldehyde and embedded in glycid ether.

Serial paraffin slides of 1–2 µm thickness were stained by H&E, the sPAS reaction, and immunohistochemistry.

### 4.5. Immunohistochemistry

Formalin-fixed and paraffin-embedded (FFPE) serial murine kidney sections 1–2 µm in thickness were stained for acetyl-CoA carboxylase, eukaryotic translation initiation factor 4E-binding protein 1 (4E-BP1), fatty acid synthase, insulin receptor substrate 1, pyruvate kinase M2, ribosomal protein S6 (RPS6), thioredoxin interacting protein, cyclin D1, snail, slug, E-Cadherin, vimentin, YAP and BrdU. FFPE human kidney sections (methods for human kidney samples are given below) were stained for ChREBP. Primary antibodies are listed in detail in Table 3. For antigen retrieval, a citrate buffer of pH 6.0 was used. Endogenous peroxidase was cleared with 1% hydrogen peroxide, and positive reactivity of primary antibodies was performed by the HRP polymer and DAB as the chromogen substrate (Dako, Glostrup, Denmark). Fatty acid synthase immunohistochemistry was conducted using an automated immunostainer (Leica Biosystems, Wetzlar, Germany) and a DAB kit.

The immunohistochemical reactions of murine tissue were assessed semi-quantitatively by comparing intensity in glycogenotic tubules or tumor with corresponding surrounding unaltered kidney tissue. Negative controls were stained without any primary antibody.

For quantification of ChREBP expression in human ccRCCs and in tubule epithelial cells of adjacent normal renal cortex, the product of staining intensity and percentage of positively stained nuclei, graded as shown in Table 4, was used as a score. For each case, one whole slide including tumor tissue and adjacent normal kidney tissue was analyzed by two independent observers. When results varied, a consensus was reached by discussion.

### 4.6. Morphologic and Morphometric Analysis

Images were acquired with a Nikon Eclipse Ni-U light microscope using the 4×/0.20, 10×/0.45 or the 20×/0.80 Plan Apo λD objective (Nikon Europe B.V., Amtstelveen, The Netherlands). CCTs were identified as circumscribed lesions of glycogen-storing tubular epithelial cells. Glycogen storage was detected by clear cytoplasm in H&E staining and a deep purple staining in PAS reaction and was verified by transmission electron microscopy (TEM) in representative sections. CCT fraction was measured by determining the area of PAS-positive staining in proximal or distal tubules using NIS Elements BR 3.2 (V2011). For each mouse, 15 randomly selected fields of view (each 22.4 mm^2^, total 336 mm^2^) were analyzed in one section per mouse. BrdU labeling index was determined as the number of BrdU-positive nuclei per 100 nuclei of tubular epithelial cells in 15 randomly selected fields of view. The number and size of preneoplastic basophilic or cystic lesions and tumors were determined in one section of each kidney per mouse. A lesion larger than 0.1 mm without necrosis was considered an adenoma, a lesion larger than 1 mm with necrosis an RCC.

### 4.7. Transmission Electron Microscopy (TEM)

Kidney tissue was fixed in 2.5% glutaraldehyde, embedded in glycid ether, and sectioned with a Leica ultratome EM UC7 (Leica Biosystems, Wetzlar, Germany) with a diamond knife. Semithin sections (500 nm and 750 nm) were stained with H&E staining, PAS reaction, and the Richardson’s staining. Ultrathin sections (70–90 nm) were stained with uranyl acetate. Images were acquired with a Libra 120 transmission electron microscope (Carl Zeiss GmbH, Oberkochen, Germany).

### 4.8. Human Renal Tissue

Renal specimens were obtained from a total of 31 kidneys that were resected for ccRCC at the Universitaetsmedizin Greifswald between 2014 and 2017. The study was approved by the local Institutional Review Board of the Universitaetsmedizin Greifswald (Greifswald, Germany; BB081/12a) and informed consent was obtained in accordance with the approval of the local Institutional Review Board of the Universitaetsmedizin Greifswald (Greifswald, Germany; BB081/12a). Our study was performed in accordance with the Declaration of Helsinki as revised in Brazil 2013.

### 4.9. Statistical Analysis

For blood glucose levels, body weight, morphometric data, and BrdU labeling index, Gaussian distribution was checked by Kolmogorov–Smirnov test or Shapiro–Wilk test. For significance testing of normally distributed data, Student’s unpaired *t*-test was used. Fishers exact test, Mann–Whitney U Test, or Wilcoxon signed-rank test was used for nonparametric data. To determine whether preneoplastic lesions occurred earlier in either of the two diabetic groups (diabetic ChREBP-KO or WT), tumor-free survival in these two groups was compared. Survival curves were made with GraphPad prism V5.01 (GraphPad Software, San Diego, CA, USA). The Gehan–Breslow–Wilcoxon test was used to determine whether the risk of event (=development of tumors) was significantly different in the two groups. For all experiments, *p*-values < 0.05 were considered statistically significant.

## Figures and Tables

**Figure 5:**
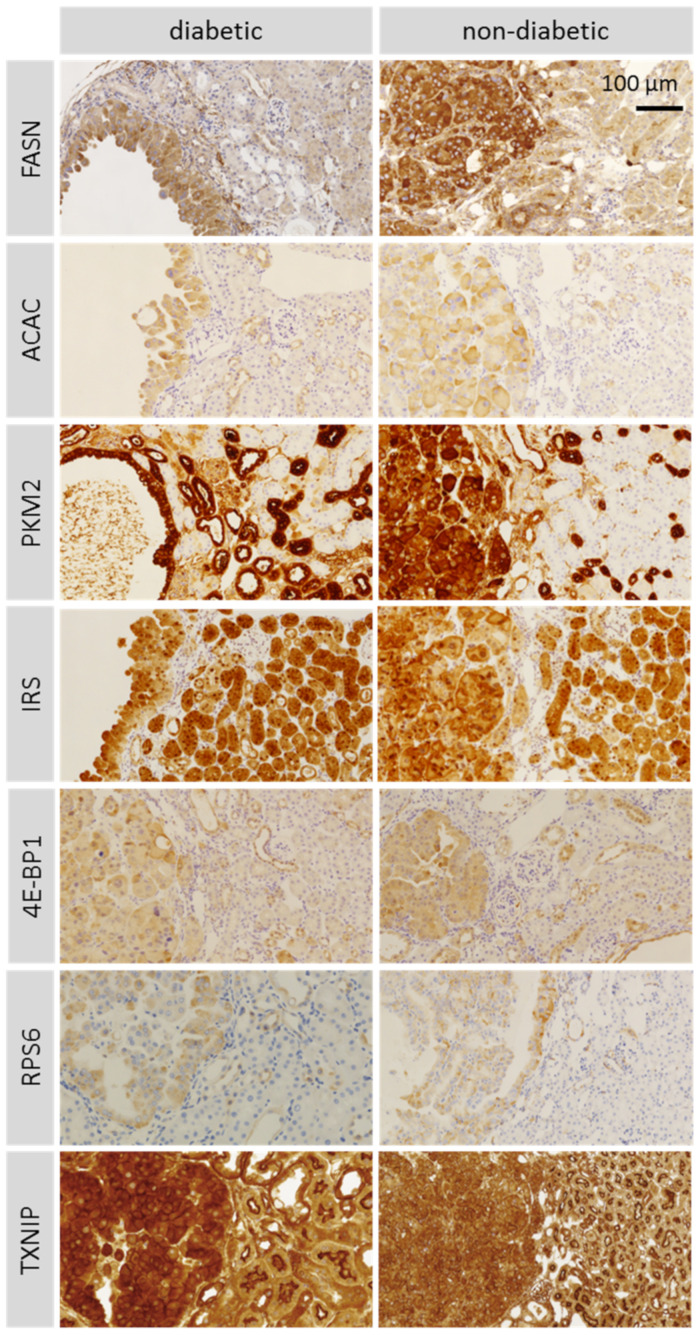
Immunohistochemical findings in tumors of diabetic and non-diabetic ChREBP-KO mice. Comparison of tumor tissue (on the left side in each picture) with adjacent kidney tissue in the representative micrographs of paraffin sections: upregulation of lipogenesis indicated by higher expression of fatty acid synthase (FASN) and acetyl-CoA carboxylase (ACAC) and an upregulation of the Akt/mTOR pathway indicated by higher expression of the downstream effectors, eukaryotic translation initiation factor 4E-binding protein 1 (4E-BP1) and ribosomal protein S6 (RPS6) in tumors. Pyruvate kinase M2 (PKM2) and insulin receptor substrate 1 (IRS) are expressed in tumors and adjacent kidney tissue. Thioredoxin interacting protein (TXNIP) is strongly expressed in the tumor cells. TXNIP positivity is restricted to the apical sides in normal tubule epithelial cells. All images are on the same scale.

**Table 1:** Classification and quantification of preneoplastic lesions and tumors in diabetic and non-diabetic WT and ChREBP-KO mice. For each group, the numbers of animals with one or more lesions fulfilling the respective criterion are given. Preneoplastic lesions were counted only if they measured ≥ 0.1 mm in diameter and showed enlarged cells with a basophilic cytoplasm or a cystic growth pattern. Lesions with necrosis measuring ≥ 1 mm in diameter were defined as renal cell carcinomas (RCCs).

Experimental Groups	ChREBP-KO, Diabetic	ChREBP-KO, Non-Diabetic	WT, Diabetic	WT, Non-Diabetic
	3 months/6 months/12 months			
Streptozotocin treatment	yes	no	yes	no
Number of animals (n) total	74	59	60	58
Number of animals 3/6/12 months (n)	40/24/10	19/19/21	25/20/15	20/18/20
At least one lesion (%)	10/25/60 ^d^	26.3 ^a^/31.6 ^b^/61.9 ^c^	4/20/100 ^d^	0 ^a^/0 ^b^/0 ^c^
At least one lesion 0.1–0.3 mm (%)	5/16.7/60	15.8/10.5/57.1	4/0/80	0/0/0
At least one lesion 0.4–0.6 mm (%)	2.5/12.5/40	21/5.3/9.5	0/5/20	0/0/0
At least one lesion 0.7–0.9 mm (%)	5/8.3/40	0/5.3/0	0/5/46.7	0/0/0
At least one lesion with necrosis (%)	0/0/0	0/0/4.8	0/0/6.7	0/0/0
At least one RCC (%)	0/0/0	5.3/15.8/4.8	0/0/13.3	0/0/0

^(a)^ *p* = 0.02, ^(b)^ *p* = 0.02, ^(c)^ *p* < 0.0001, ^(d)^ *p* = 0.02.

**Table 2:** Experimental groups.

Age	1 Week		4 Weeks		3 Months		6 Months		12 Months	
Diabetes	yes	no	yes	no	yes	no	yes	no	yes	no
Wild type	*n* = 9	*n* = 10	*n* = 7	*n* = 10	*n* = 25	*n* = 20	*n* = 21	*n* = 19	*n* = 17	*n* = 20
ChREBP-KO	*n* = 11	*n* = 10	*n* = 7	*n* = 11	*n* = 40	*n* = 19	*n* = 25	*n* = 20	*n* = 10	*n* = 21

**Table 3:** Primary antibodies. ON = overnight incubation.

Protein	Dilution	Species	Company
ACAC	1/200	Rabbit	Cell Signaling, Danvers, MA, USA
BrdU	1/50 ON	Mouse	Dako, Hamburg, Germany
ChREBP	1/400	Rabbit	Novusbio, Littleton, CO, USA
4E-BP1	1/600	Rabbit	Cell Signaling, Danvers, MA, USA
FASN	1/400 ON	Mouse	BD biosciences, San Diego, CA, USA
IRS1	1/400	Rabbit	Abcam, Cambridge, UK
PKM2	1/100	Rabbit	Cell Signaling, Danvers, MA, USA
RPS6	1/300	Rabbit	Cell Signaling, Danvers, MA, USA
TXNIP	1/1600	Rabbit	Abcam, Cambridge, UK
Cyclin D1	1/100	Rabbit	Epitomics, Burlingame, CA, USA
Snail	1/100	Rabbit	Abcam, Cambridge, UK
Slug	1/100	Rabbit	Abcam, Cambridge, UK
E-Cadherin	1/100	Mouse	BD biosciences, San Diego, CA, USA
Vimentin	1/100	Mouse	Santa Cruz, Santa Cruz, CA, USA
YAP	1/100	Rabbit	Cell Signaling, Danvers, MA, USA

**Table 4:** Scoring of immunohistochemical staining for ChREBP on human FFPE tissue.

Percentage of positively stained nuclei	1	<10%
	2	10–50%
	3	51–80%
	4	>80%
Staining intensity	0	no staining
	1	weak staining
	2	moderate staining
	3	strong staining

## Data Availability

All relevant data that support the findings of this study are available in the main text or the Supplementary Materials. For general comments or material requests, please contact the corresponding author.

## Supplementary Materials

The following supporting information can be downloaded at: https://www.mdpi.com/article/10.3390/ijms252111438/s1.
