# Supplementary material for: Knockout of the Carbohydrate Responsive Element Binding Protein Enhances Proliferation and Tumorigenesis in Renal Tubules of Mice
Source: Int J Mol Sci. 2024 Oct 24;25(21):11438. doi: 10.3390/ijms252111438 (PMC11545909; doi:10.3390/ijms252111438)
Supplement: Supplementary file 1 [file ijms-25-11438-s001.zip › ijms-3226674-supplementary.pdf]

Supplement

Supplementary Figure S1

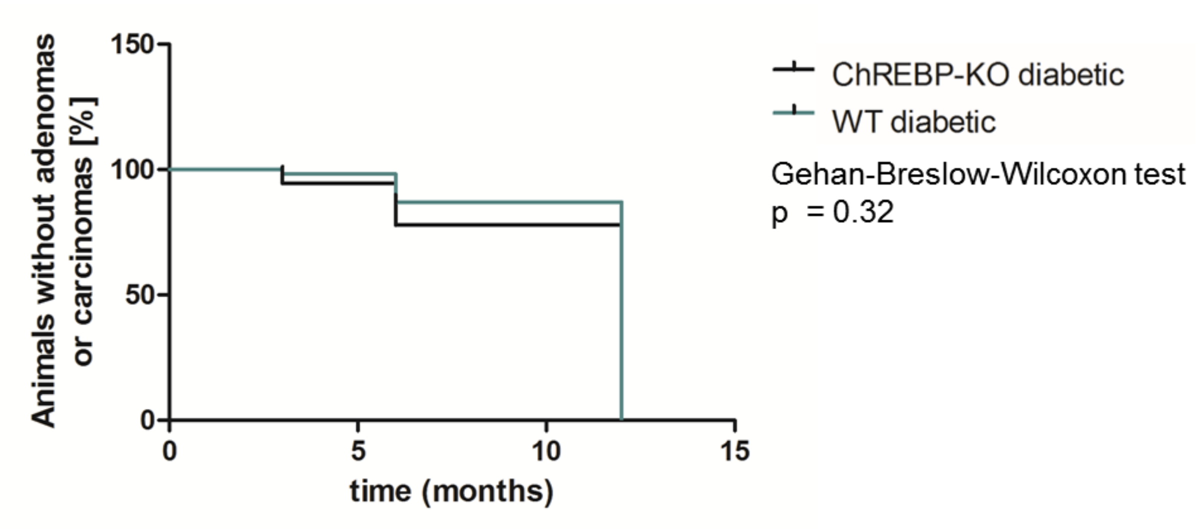

Supplementary figure S1 Numbers of mice without tumors in diabetic WT vs. diabetic ChREBP-KO group.

Supplementary Figure S2

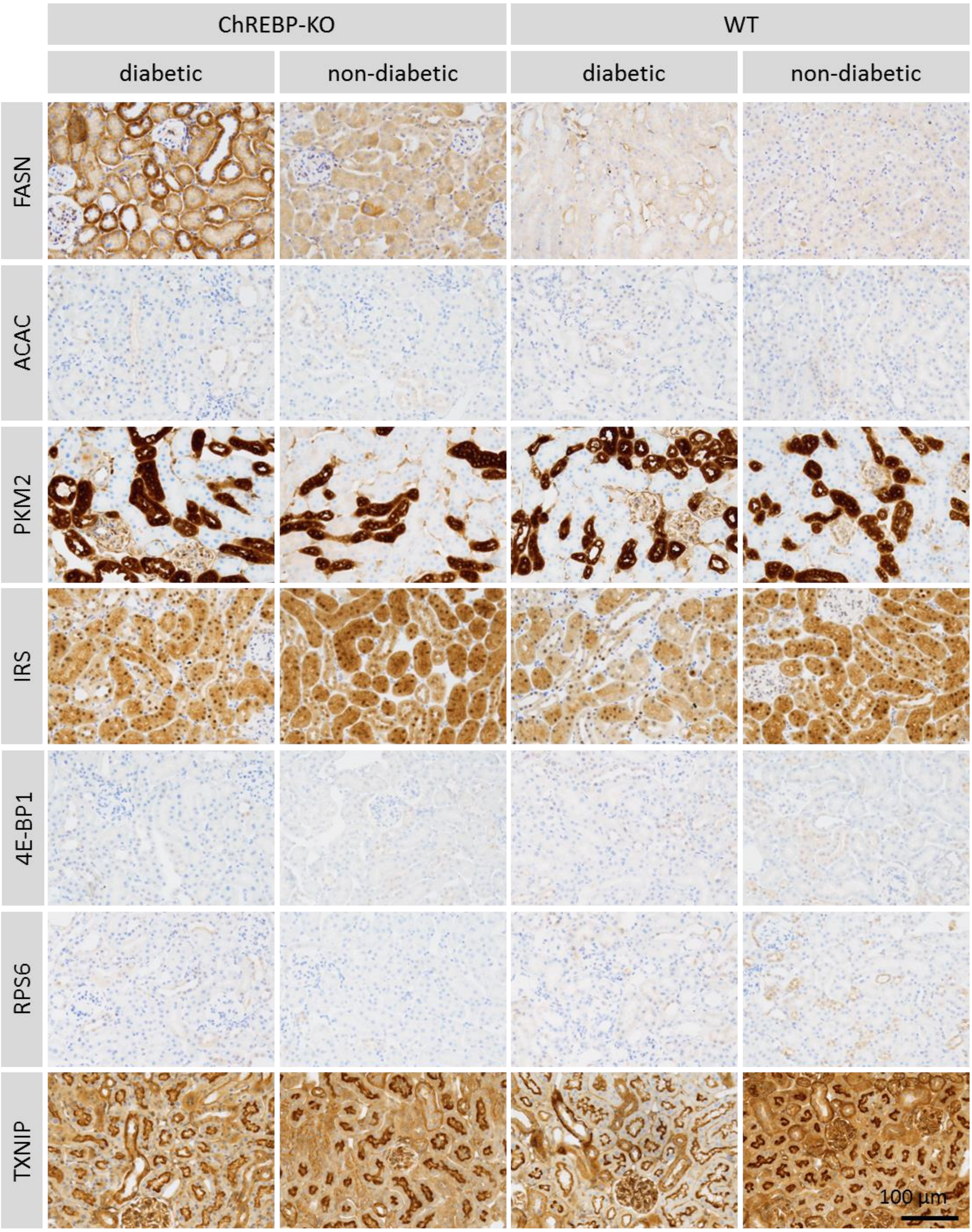

Supplementary figure S2 Immunohistochemical findings in normal kidney tissue of diabetic and non-diabetic ChREBP-KO and WT mice. Paraffin sections from mice of each genotype were stained on the same slide. Note increased expression of fatty acid synthase (FASN) in the kidney tissue of ChREBP-KO mice compared to WT. There were no differences in expression of acetyl-CoA carboxylase (ACAC), pyruvate kinase M2 (PKM2), insulin receptor substrate 1 (IRS), eukaryotic translation initiation factor 4E-binding protein 1 (4E-BP1), ribosomal protein S6 (RPS6) and thioredoxin interacting protein (TXNIP) in normal kidney tissue between the two genotypes.

Supplementary Figure S3

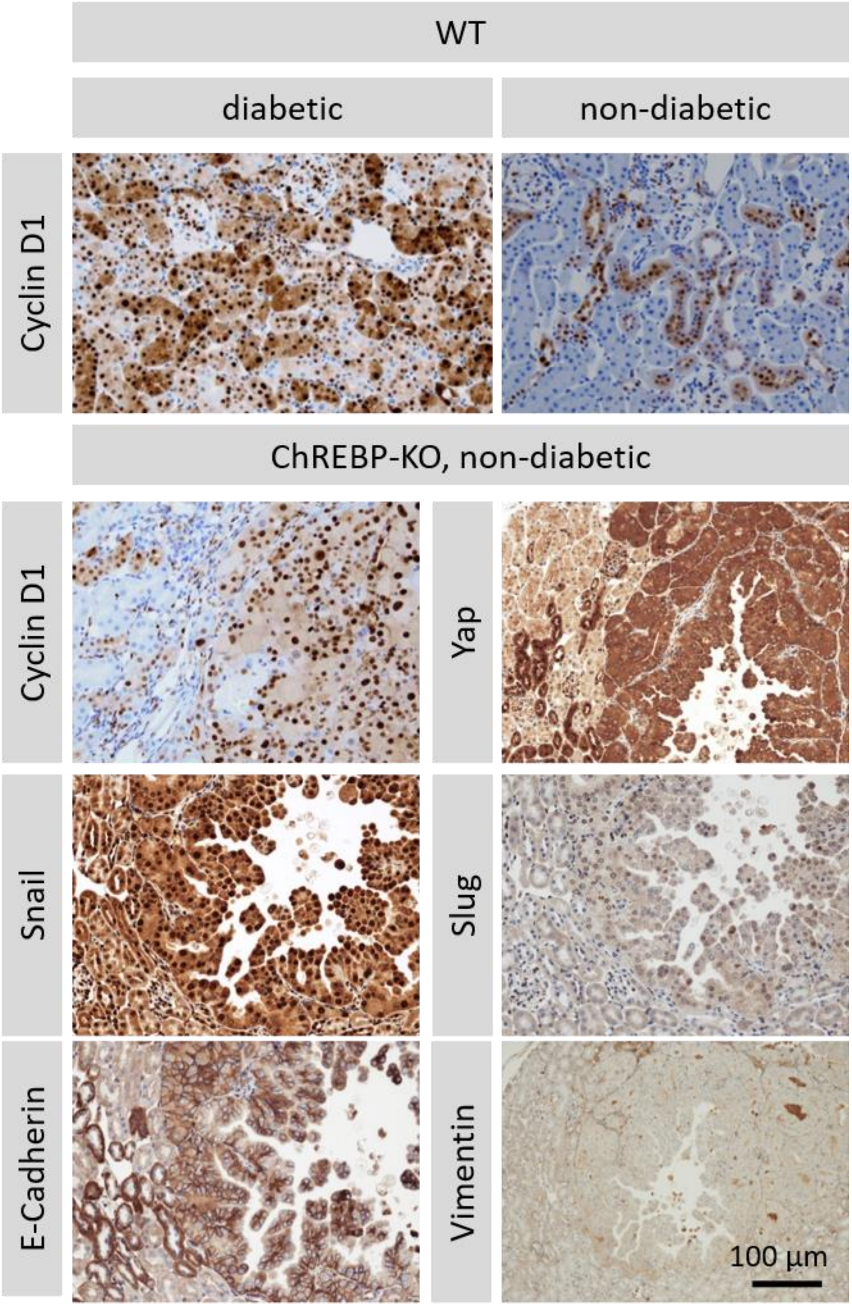

Supplementary figure S3 Additional immunohistochemistry data. An upregulation of cyclin D1 was seen in kidney tissue of diabetic WT mice as well as in kidney tumors of non-diabetic ChREBP-KO mice, contrasting lower cyclin D1 expression in normal kidney tubules of WT controls. Renal tumors of ChREBP-KO mice showed strong cytoplasmatic, but not nuclear YAP positivity. The epithelial-mesenchymal transition markers snail, slug and vimentin were not upregulated in tumors compared to adjacent unaltered kidney tissue, while E-cadherin expression was present. All images are on the same scale.

Supplementary Figure S4

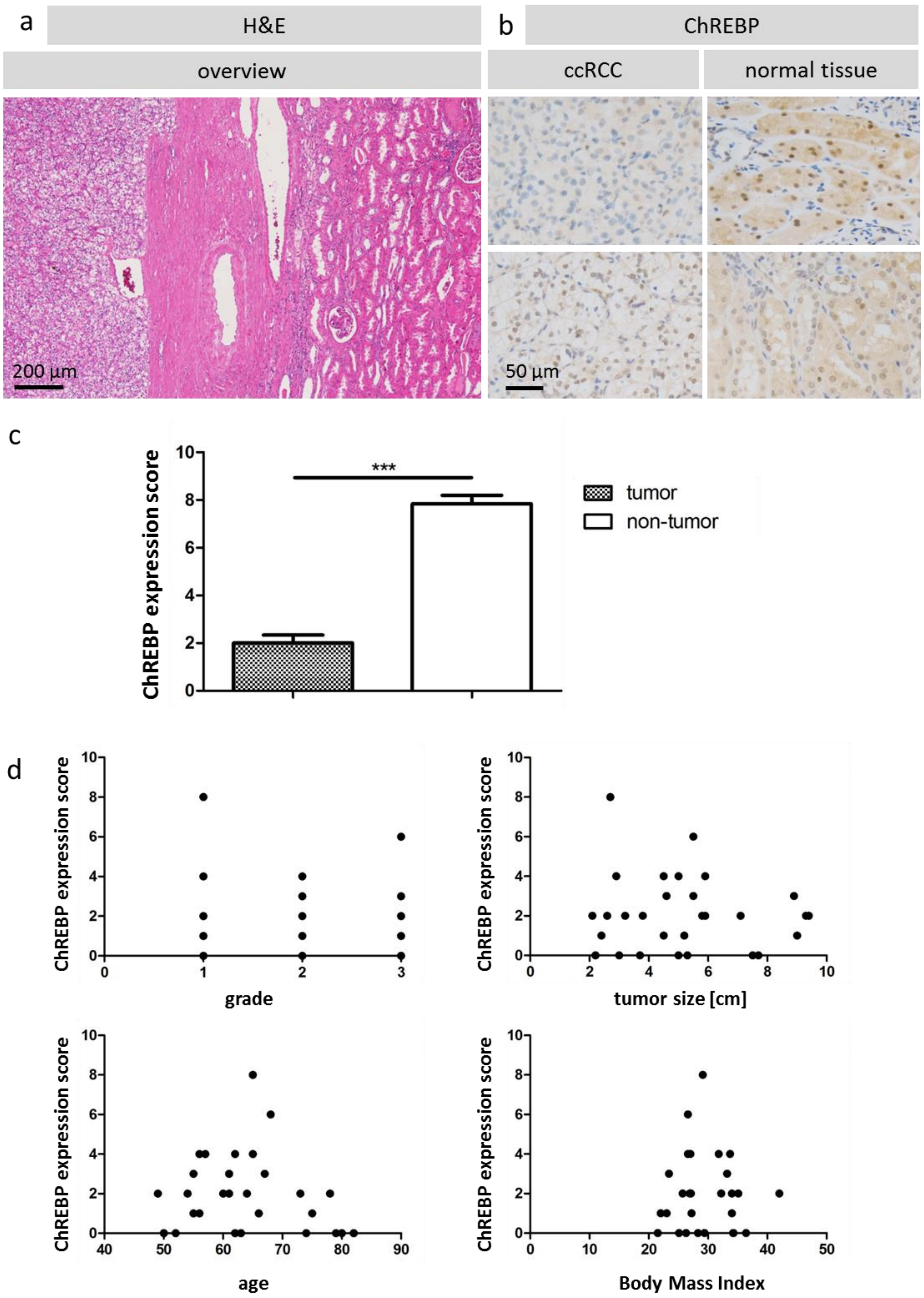

Supplementary figure S4 ChREBP expression in ccRCC in humans. (a) Tumors (left side) in H&E staining. (**b)** ChREBP expression in ccRCC was assessed by immunohistochemistry on paraffin sections and was scored according to nuclear staining intensity and percentage of positive cells. The micrographs on the left side of the column show ccRCC tissue and the micrographs on the right side

show corresponding normal kidney tissue on the same slide. The images in the first row exemplary show reduced ChREBP expression in the tumor: Tumor was scored as 2 x 1 = 2, meaning weak ChREBP expression in 10 - 50 % of cells. Normal tissue was scored as 4 x 3 = 12, meaning strong ChREBP expression in > 80 % of tubule epithelial cells. The images in the second row give an example of unaltered ChREBP expression in the tumor: Tumor and normal tissue were both scored as 4 x 2 = 8, meaning intermediate ChREBP expression in > 80 % of cells. **(c)** The graph shows ChREBP expression scores in n = 31 cases of ccRCC. *p*-value < 0.001 is indicated by three asterisks. **(d)** The graphs show ChREBP expression scores of n = 31 cases of ccRCC and corresponding WHO/ISUP grade, tumor size, patient's age and patient's body mass index; which did not correlate with ChREBP expression.
